# Traditional machine learning in biomedical image analysis: before you go too deep

**DOI:** 10.3389/frai.2026.1695230

**Published:** 2026-01-29

**Authors:** Elizaveta Chechekhina, Nikita Voloshin, Maksim Solopov, Pyotr Tyurin-Kuzmin, Konstantin Kulebyakin

**Affiliations:** 1 Medical Research and Educational Institute, Lomonosov Moscow State University, Moscow, Russia; 2 V.K. Gusak Institute of Emergency and Reconstructive Surgery, Donetsk, Russia

**Keywords:** biomedical image analysis, object classification, radiomics, semantic segmentation, traditional machine learning

## Abstract

Traditional machine learning (TML) algorithms remain indispensable tools for the analysis of biomedical images, offering significant advantages in multimodal data integration, interpretability, computational efficiency, and robustness on smaller datasets. This review provides a comprehensive examination of TML applications across a broad spectrum of biomedical imaging modalities, highlighting its core principles, practical implementation, and unique benefits in the era of deep learning (DL). We outline the fundamental concepts of machine learning and describe key biomedical imaging tasks successfully addressed by TML. We also highlight the most popular platforms, which empower clinicians and researchers to utilize TML. DL now dominates many areas of medical image analysis due to superior performance and end-to-end feature learning. Using the most prominent examples, we analyze how TML retains unique value for applications with multimodal data processing, limited data, interpretability requirements, or rapid prototyping needs. Supported by increasingly democratized tools and validated by robust clinical studies, TML remains a vital methodology for extracting quantitative and qualitative insights from biomedical image data, ensuring its continued relevance in both research and clinical practice.

## Introduction

By 2025, deep learning (DL) has achieved remarkable progress in biomedical imaging, with vision large language models (vLLMs) now setting new standards for automated interpretation and analysis (Li et al., 2023; Lan et al., 2025). Yet, despite the complexity and high competence of these modern approaches, much earlier and simpler traditional machine learning (TML) methods remain not only in use but actively thrive. For example, according to Dimensions citation data available via Altmetric, the ImageJ WEKA trainable segmentation paper has accumulated more than 2,000 citations overall, with more than 800 of them appearing in just the last 2 years, reflecting sustained growth of ImageJ WEKA usage in recent biomedical and microscopic imaging studies. Importantly, the field-classification of these citing articles is dominated by “Biomedical and Clinical Sciences” and “Biological Sciences,” indicating that Trainable Weka Segmentation is used primarily in biological imaging and clinically oriented workflows rather than in generic computer vision contexts. A similar pattern is observed for PyRadiomics, whose foundational radiomics toolbox paper has surpassed about 6,000 citations in total, with the majority also concentrated in the most recent few years, underscoring its status as a de facto standard for clinical radiomics feature extraction workflows. Here again, Dimensions category data show that most citing publications fall under “Biomedical and Clinical Sciences” and “Biological Sciences,” consistent with the strong focus of radiomics on oncology, imaging biomarkers, and translational medical research. These citation trajectories themselves suggest that interpretability and robustness of handcrafted features retain high practical value in current biomedical imaging research.

Why do these straightforward, interpretable methods continue to attract such attention and widespread adoption? What makes TML approaches still relevant in the era of deep learning? Existing research in this field is very broad, but it is mostly dedicated to comparison of TML and DL in general. At the same time biomedical imaging is a narrow field with specific requirements, and there is a lack of understanding how TML manages to outperform DL in this particular area.

This mini-review aims to fill a notable gap in guidelines that address conditions specific to biomedical imaging where TML is particularly advantageous. Here, decision-making factors such as limited dataset size, hardware constraints, and the need for biological interpretability are reviewed to clarify when TML may be the more suitable choice. We outline the ‘middle-ground’ scenarios of biomedical data processing in which TML is not merely an alternative but the most advisable approach.

Our mini-review is structured as follows. First, we outline the fundamental advantages of TML. Next, we illustrate these advantages with relevant examples of object classification with Radiomics and semantic segmentation with ImageJ WEKA. In defining the scope of this work, we focus on peer-reviewed studies in biomedical and clinical imaging that apply traditional machine learning to handcrafted features or classical pixel-wise classifiers, having screened recent literature in major scholarly databases and excluded purely methodological or synthetic-benchmark papers that lack biological or clinical context. Finally, we discuss the role of TML within the contemporary image analysis ecosystem, highlighting practical decision rules for choosing between TML, DL, and hybrid approaches.

### Advantages of traditional machine learning

TML has emerged as a foundational approach for extracting quantitative and qualitative insights from biomedical images across diverse modalities, offering unique advantages that remain highly relevant in the era of DL. The application of TML spans multiple imaging modalities including microscopy, radiography, computed tomography (CT), magnetic resonance imaging (MRI) and ultrasound.

A key advantages of TML include:

#### Interpretability

The fundamental distinction between TML and DL lies in the feature extraction process. While DL models automatically learn features from raw data, TML requires explicit feature engineering and extraction as preprocessing steps. This characteristic, often perceived as a limitation, actually provides significant advantages in medical applications where interpretability and clinical validation are paramount (Hill, 2024).

#### Computational efficiency

TML models require fewer resources than DL algorithms, facilitating deployment on standard central processing unit (CPU) hardware in resource-limited settings. Rapid training and inference enable real-time applications in point-of-care environments (Kaur et al., 2019).

#### Performance on limited data

In tasks with small datasets—common in biomedicine—TML algorithms can outperform DL models (Chang et al., 2023). This is because these methods are less complex and have fewer parameters, which reduces their propensity to overfit in situations where training data is limited (Ying, 2019). Furthermore, they can achieve robust performance with hundreds of samples, unlike thousands in DL methods (Silvey and Liu, 2024).

#### Multimodal data integration

TML uniquely accommodates hybrid feature spaces by combining image-derived features (e.g., radiomic texture from MRI/CT), clinical metadata (lab results, patient history), molecular data (genomic/proteomic markers), and other data (Xu et al., 2024). For deep learning algorithms integration of image data with different data modalities requires special algorithm architectures (Stahlschmidt et al., 2022). Only vLLMs can match TML algorithms in this aspect, as they naturally allow a mixture of image and text data as an input (Saab et al., 2024).

#### Potential regulatory compliance

Rule-based algorithms, in contrast to machine learning ones, are easier to regulate due to higher transparency (Hill, 2024). While explainability of TML methods is far from complete, they are much closer to rule-based algorithms than deep learning methods. Models can be audited feature-by-feature, better satisfying strict medical device regulations.

## TML for image analysis: basics of inner mechanics

**Figure 1:**
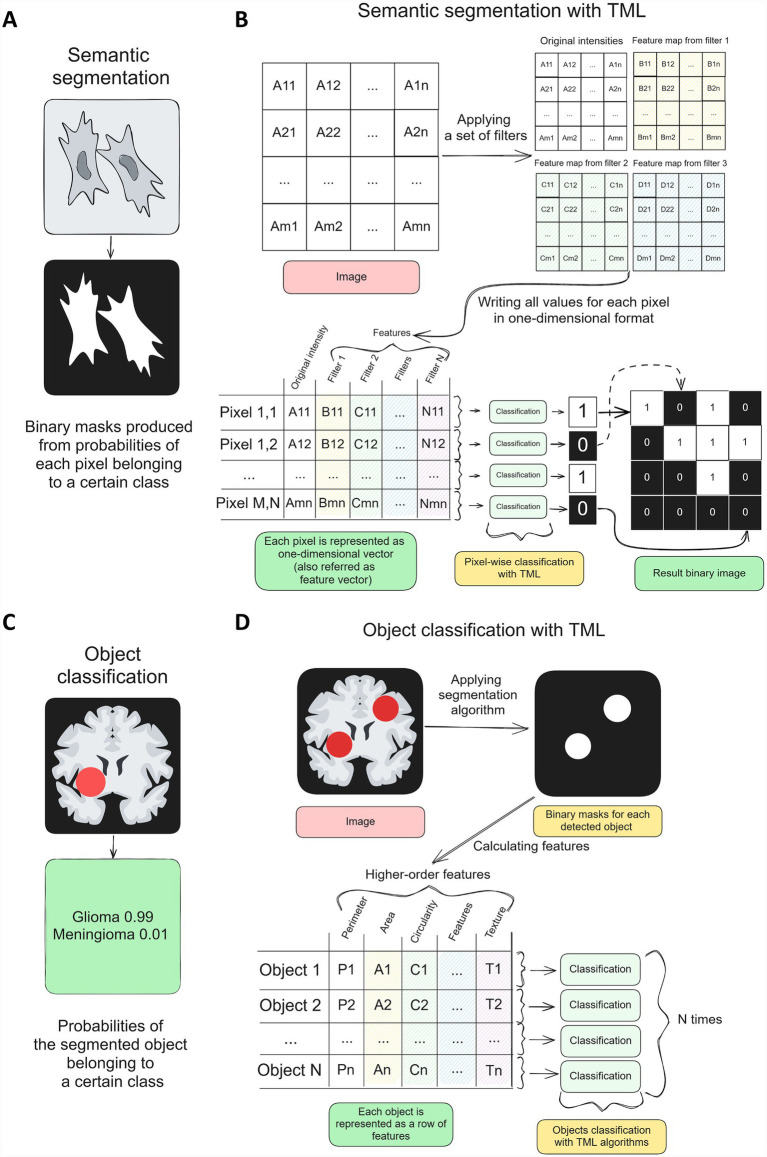
Two typical tasks in biomedical image analysis solved with TML algorithms. **(A,B)** Semantic segmentation—pixel-wise features extracted via image filters are classified into binary masks. **(C,D)** Object classification, where segmented objects are characterized by extracted features and subsequently classified using TML algorithms.

TML algorithms remain highly relevant in biomedical image analysis, offering robust solutions across a wide spectrum of imaging modalities. Below, we outline two main modalities of tasks where TML algorithms are successfully applied in biomedical image analysis (Figures 1A,C):

- Semantic segmentation (or pixel classification), i.e., predicting whether each pixel on an image belongs to some class thus producing a binary mask (Figures 1A,B; Ghosh et al., 2019). Semantic segmentation is used in cases where there is a need to select some objects or regions in images for consequent objects counting, area calculations and other relevant information extraction.
- Object classification, i.e., predicting whether an object or region on an image belongs to a certain class (Lugnan et al., 2020). Typically it is done by segmenting an object of interest first, then by extracting its features by some algorithm (see below description of PyRadiomics). The extracted set of features is classified by a TML algorithm to yield a label for the object (see Figures 1C,D).

### Semantic segmentation

The main feature of TML algorithms is that they take as input a feature vector—a set of parameters for each individual object, represented as a one-dimensional array. In case of semantic segmentation, each pixel of an image is converted into a feature vector and then classified (thus the alternative name pixel classification) to yield a binary mask (see Figure 1B). To increase the information about each pixel, a set of filters is usually applied to a whole image and values from result maps are used as features; examples of such filters are Gaussian, Laplassian, Gabor, Mean, Median filters etc. (see Figure 1B). This process is called feature extraction, since each filter highlights different features of objects on a picture such as edges, intensity peaks and others. Method of image segmentation through feature extraction with filters and pixel classification is especially popular in microscopy (cell culture studies, histopathology, material sciences etc.). It can be done in practice with ImageJ Trainable WEKA Segmentation plugin (Arganda-Carreras et al., 2017), ilastik (Berg et al., 2019), QuPath (Bankhead et al., 2017), or Napari (Ahlers et al., 2023) plugins.

### Object classification

Another approach to image vectorization is extracting higher-order features from objects to classify them (Figure 1D). It is done by segmenting objects first [with Segment Anything Models significantly simplifying that process (Kirillov et al., 2023; Ma et al., 2024; Archit et al., 2025)] and then extracting features from them such as shape, intensity, and texture features. CellProfiler (Carpenter et al., 2006) is a software that focuses on feature extraction for microscopy studies, while in radiology *radiomics* is a general term for a set of tools for extracting relevant features from objects on different diagnostic images (Kumar et al., 2012).

## Relevant examples of TML using in practice

After outlining the main biomedical image analysis task categories that TML techniques address and their theoretical underpinnings, it should be noted that these techniques are still very applicable in current research. They continue to be widely used in many different fields due to their interpretability and robustness. In the sections that follow, we provide real-world examples that demonstrate the usefulness and long-term effects of these established methods.

### Radiomics: when traditional machine learning works better

Radiomics has emerged as one of the most dynamic and clinically relevant fields for the application of TML in biomedical image analysis. The core principle of radiomics involves extracting quantitative features from medical images—such as CT, MRI, or ultrasound—and utilizing TML algorithms to construct predictive models for diagnosis, prognosis, and treatment response (Wagner et al., 2021; Cè et al., 2024; Majumder et al., 2024).

Despite the growing adoption of deep learning in radiomics, TML maintains its clinical utility. Traditional radiomics features are explicitly defined and extracted on the basis of predetermined mathematical formulas, offering intrinsic interpretability that allows clinicians to understand which specific characteristics contribute to diagnostic decisions. This transparency contrasts sharply with deep learning’s “black box” nature, where the learned feature extractors remain largely opaque to clinical interpretation (Wang et al., 2025). Recent studies have demonstrated that TML-based radiomics pipelines not only remain competitive with deep learning but frequently outperform them when datasets are small, interpretability is required, or clinical implementation is the primary objective (Chang et al., 2023).

Concrete quantitative evidence of TML advantages under specific conditions is demonstrated by a comparative study of liver tumor differentiation using MRI data. In this study, an SVM-based radiomics model achieved an AUC of 0.879 on the test set, while a DenseNet-based deep learning model showed significantly lower performance with an AUC of 0.717 (Du et al., 2022). The statistically significant difference (*p* < 0.001) confirms the superiority of the traditional approach in this context. The authors attribute this to the fact that the radiomics model utilized only 8 carefully selected features from 1,049 possible ones, while the deep learning model processed volumes of 100 × 100 × 100 voxels (1,000,000 features), which, combined with the limited sample size (426 training samples), led to overfitting and reduced generalization capability.

In each case, specialists should select a set of features to extract from objects of interest. But there are hundreds of possible features and it’s a challenge to find the key ones. To alleviate that, automated frameworks were created that perform this selection of important features from a large set. Such automated frameworks have streamlined radiomics model construction and validation, reducing manual trial-and-error and improving reproducibility across diverse clinical tasks. The WORC framework, validated across 12 clinical applications, has demonstrated superior performance compared to both basic radiomics baselines and human expert approaches (Starmans et al., 2025). This framework addresses the critical challenge of method selection by automatically optimizing the entire radiomics workflow, from preprocessing through feature extraction to classification algorithm selection.

Similarly, the Simplatab framework represents an advancement in automated machine learning for radiomics-based clinical applications. Evaluated on a large pan-European cohort of 4,816 patients from 12 clinical centers across nine countries, Simplatab integrates comprehensive functionality including data bias detection, feature selection, model training with hyperparameter optimization, and explainable AI analysis (Zaridis et al., 2025). The framework’s user-friendly interface requires no coding expertise while providing detailed performance reports and robust bias assessment in human-understandable formats.

In the multimodal study by Xu et al. (2024), researchers combined radiomic features from multiparametric MRI with automatically extracted pathomorphological features (using CellProfiler (McQuin et al., 2018)) and clinical patient data—including tumor stage, biomarker levels (e.g., CA-125), and treatment history. This integration yielded an improvement in prognostic accuracy (94%) compared to unimodal approaches. In another study, comparative research in lung and thymic tumor imaging shows that TML-based radiomics can exceed or match deep learning models, particularly in heterogeneous or rare disease cohorts, where supplementing imaging features with clinical variables like smoking history and comorbidity profiles significantly boosted model robustness against dataset shifts (Chang et al., 2023). These researches highlight the major advantage of TML algorithms—seamless integration of image-derived data with different data modalities, which is a challenge for deep learning algorithms (Stahlschmidt et al., 2022).

Table 1 summarizes several recent, high-impact studies highlighting the versatility and effectiveness of TML in radiomics.

**Table 1:** Recent studies demonstrating successful applications of TML algorithms in radiomics.

Study and year	Imaging modality/Task	TML approach and outcome
Automated ML framework for radiomics (WORC), 2025	12 clinical tasks (CT, MRI, etc.)	AutoML with TML (XGBoost, SVM, RF); outperformed manual pipelines and human experts; improved reproducibility (Starmans et al., 2025)
Simplatab framework, 2025	Bi-parametric MRI, clinically significant prostate cancer	Automated ML framework with XAI integration; comprehensive bias detection and model vulnerability assessment (Zaridis et al., 2025)
Decoding Radiomics: ML workflow guide, 2024	Step-by-step radiomics workflow	Comprehensive review; emphasizes feature extraction, selection, and TML classifier choice for robust clinical models (Cè et al., 2024)
AutoML radiomics for pulmonary nodules, 2024	CT chest, nodule chronicity prediction	Ensemble model: sensitivity 0.65, specificity 0.92, AUC 0.88; outperformed individual radiologists (Mehta et al., 2024)
Prediction of the efficacy of neoadjuvant chemotherapy in breast cancer, 2024	MRI + histopathology images	Different models: sensitivity 0.37–0.88, specificity 0.69–0.91, AUC 0.65–0.91 (Xu et al., 2024)
Differentiation of thymic epithelial tumors, 2023	Lung CT	Feature selection + RF, XGBoost, CatBoost, etc.; TML outperformed DL in small datasets; >90% accuracy (Chang et al., 2023)
Multi-view SVM + for liver cancer, 2021	Ultrasound (B-mode, CEUS), liver cancer	Multi-kernel SVM + using multi-phase features; accuracy 88.2%, sensitivity 87.0%, specificity 89.4% (Zhang et al., 2021)

Current evidence demonstrates that TML-based radiomics consistently achieves competitive or superior performance compared to deep learning in small-to-moderate dataset scenarios, particularly when multimodal data integration and clinical interpretability are prioritized. Automated frameworks such as WORC and Simplatab have validated TML’s robustness across multiple clinical applications. However, standardized benchmarking protocols comparing TML and DL across diverse imaging modalities remain lacking, and optimal feature selection strategies for highly heterogeneous cohorts require further investigation.

### Traditional machine learning is convenient and fast alternative to deep learning for semantic segmentation: ImageJ WEKA example

If segmentation on some image or set of images is required, the simplest approach is manual segmentation or threshold-based segmentation. It works perfectly for cases with small amounts of simple objects with high contrast. The second option is deep learning, which suits cases with a large number of images and objects with complex shapes and low contrast. But it needs dataset creation, where the user should spend a substantial amount of time to manually draw masks on a set of images. It also requires a graphics processing unit (GPU) for training and inference. In its turn, TML for segmentation perfectly fits the gap between manual or threshold-based segmentation and deep learning. Let us examine this further using ImageJ WEKA as an example—a trainable segmentation plugin (Arganda-Carreras et al., 2017), which is one of the most popular tools for segmenting images by pixel-wise classification with TML.

Firstly, the ImageJ WEKA trainable segmentation plugin does not require a GPU to run. Secondly, it requires significantly less data to train in comparison to deep learning: sometimes a few labeled pixels on a single image that take seconds to draw is enough. It can be viewed as an extension to manual segmentation: instead of segmenting all regions manually on an image, the user labels only a small portion, while the plugin completes these labels to the whole image. In that extent it is similar to Segment Anything Models (Kirillov et al., 2023): they too complete user prompts to masks, but for an instance segmentation task, where separating individual objects is the priority. Thirdly, it is easily tunable: users can adjust a set of filters for feature extraction and TML algorithm for pixel classification making it suitable for a wide range of use cases; at the same time deep learning segmentation algorithms have much less hyperparameters to tune at the inference stage without involvement of training algorithms. Finally, it is integrated into ImageJ (Schindelin et al., 2012; Schneider et al., 2012), one of the most popular tools for image processing with robust functionality, which makes it even more convenient for pre- and postprocessing of images.

Thus, ImageJ WEKA trainable segmentation successfully fills the gap between the most convenient manual or threshold-based segmentation and demanding deep learning methods. It is best suited for cases with medium or large amounts of data, where there is no requirement of separating densely located individual objects with complex shapes. Among the most recent examples, ImageJ WEKA trainable segmentation was successfully used to discern vessels from spheroids and background by dual-channel phase-GFP images with a relatively small training dataset of 28 images (Wong et al., 2025). In another recent paper, it was used for the relatively simple task of discerning cells from background on fluorescent images to quantify the gaps and assess cell migration (Bischoff et al., 2025). At the same time it’s not the only player in the field of TML for semantic segmentation. This functionality is also included in such tools as ilastik (Berg et al., 2019), QuPath (Bankhead et al., 2017) and various Napari (Ahlers et al., 2023) plugins.

Evidence confirms that ImageJ WEKA and similar TML-based segmentation tools successfully address “middle-ground” use cases, requiring minimal training data and computational resources while maintaining adequate accuracy for moderately complex segmentation tasks. Nevertheless, systematic comparative studies quantifying performance trade-offs between TML-based pixel classification and modern foundation models across diverse biological imaging contexts are scarce. Best practices for filter selection and hyperparameter tuning in TML segmentation workflows also remain largely empirical.

## Discussion and conclusions

Deep learning is rapidly transforming biomedical image analysis, enabling unprecedented advances in image segmentation, classification, and feature discovery (Shen et al., 2017; Haque and Neubert, 2020; Ben Yedder et al., 2021). Its capacity to automatically learn complex, hierarchical representations from raw data has opened entirely new horizons for precision diagnostics, personalized medicine and biomedical research. However, these remarkable capabilities come at a price: deep learning models are inherently complex, computationally demanding, and often require large, well-annotated datasets for robust training. Their “black box” nature also presents significant challenges for clinical interpretability and reliability, particularly in high-stakes medical settings (Luo et al., 2019; Salahuddin et al., 2022; ŞAHiN et al., 2025).

At the same time, there are numerous clinical and research scenarios where such complexity is unnecessary or even counterproductive. Many diagnostic and prognostic tasks involve well-understood imaging biomarkers or operate in data-limited environments—contexts in which the interpretability, efficiency, and lower data requirements of TML approaches offer clear advantages. In these cases, the sophistication of deep learning may be redundant, introducing additional barriers without substantial gains in performance or clinical value. The same applies to biomedical research: deep learning is the “go-to” method for complex tasks, but there are a lot of scenarios where a simpler TML approach is more efficient.

**Figure 2:**
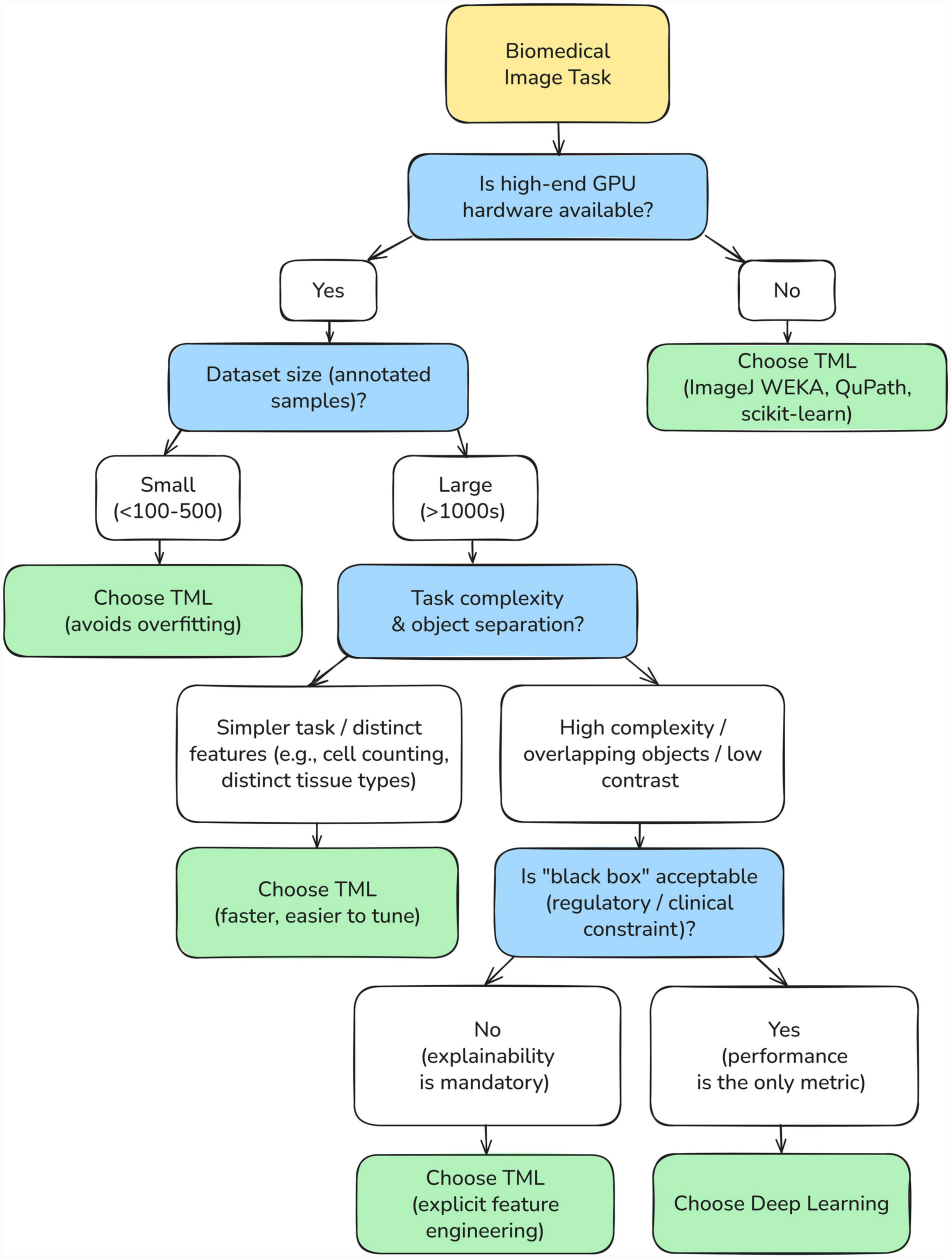
Decision scheme for selecting between TML and DL in biomedical image analysis.

To guide researchers in navigating these trade-offs, we summarize the decision-making process in Figure 2. As illustrated, TML remains the optimal strategy in contexts defined by limited computational resources, small dataset sizes, or strict requirements for biological interpretability.

TML thus occupies a unique and increasingly important niche in biomedical image analysis. It excels in the “middle ground”—tasks that are too complex for manual or rule-based methods, yet do not justify involvement of deep neural networks. Importantly, TML-based tools are not a legacy approach—it is an actively developing field that continues to deliver innovation. For example, the integration of radiomic features with pathomorphological and clinical data, the advent of automated machine learning platforms, and ongoing advances in feature standardization (Zwanenburg et al., 2020; Whybra et al., 2024) all underscore the vitality of this field.

Thus, TML offers significant advantages over deep learning methods in the field of biomedical image processing. It offers better interpretability, possibility of multimodal data integration, and often performs better on limited data with less computational demands, which are crucial features for both medicine and biological research. TML bridges the gap between manual analysis and the complexities of deep learning, and ensures that image analysis in biomedicine remains accessible, interpretable, and impactful across diverse scenarios.

## Future directions

The ongoing evolution of TML in biomedical imaging opens several promising directions for future development. Hybrid TML-DL architectures represent a particularly compelling way, where deep learning serves as an automated feature extractor while TML classifiers maintain interpretability and multimodal integration capabilities. Continued maturation of standardization initiatives, exemplified by the Image Biomarker Standardization Initiative (IBSI), will ensure that TML-based radiomics features remain reproducible across institutions, scanners, and imaging protocols, with extension to modalities beyond radiology such as microscopy and ultrasound (Zwanenburg et al., 2020; Whybra et al., 2024). Rapidly advancing AutoML frameworks for TML may soon incorporate federated learning capabilities for collaborative model development across clinical sites and integrate explainable AI modules to facilitate regulatory approval and clinician trust (de Vries et al., 2023; Ali et al., 2024; Raza et al., 2025; Singh et al., 2025). The underexplored strength of TML in multimodal data fusion warrants systematic investigation. This investigation should determine optimal strategies for integrating imaging-derived features with genomics, proteomics, electronic health records, and patient-reported outcomes. The goal is to yield more holistic and personalized diagnostic models. Finally, as vLLMs continue to advance, their potential synergy with TML should be explored. A promising integration strategy could involve vLLMs generating rich semantic descriptions of medical images, which TML classifiers could then combine with traditional radiomics features and clinical metadata to create interpretable yet powerful diagnostic pipelines.
